# The Immune Biology of the Adrenal Gland Microenvironment and Its Role in Metastatic Progression

**DOI:** 10.3390/ijms27031153

**Published:** 2026-01-23

**Authors:** Natalie M. Liu, Cyrus J. Sholevar, Makan Karimzadeh, Jay Uppuluri, Clemens Van Dongen, Claire E. Graves, Michael J. Campbell, Anthony E. Zamora, Sean J. Judge, Robert J. Canter

**Affiliations:** 1 Division of Surgical Oncology, Department of Surgery, UC Davis Medical Center, University of California Davis, Sacramento, CA 95817, USA; namliu@health.ucdavis.edu (N.M.L.);; 2 Department of Internal Medicine, School of Medicine, University of California Davis, Davis, CA 95616, USA

**Keywords:** adrenal gland, adrenal metastases, immunotherapy, tumor microenvironment

## Abstract

Metastatic lesions are the most common malignant tumor of the adrenal gland. While surgery can have a favorable surgical outcome for isolated adrenal metastatic lesions, most adrenal metastases occur in the context of disseminated disease, and the overall prognosis remains poor. Although data are limited, metastatic lesions from diverse solid tumors to the adrenal gland have typically demonstrated poor response to immunotherapy, particularly immune checkpoint inhibitors with programmed cell death protein 1 (PD-1)/programmed death-ligand 1 (PD-L1) blockade. This apparent resistance to immunotherapy suggests that the adrenal gland microenvironment may be influenced by local microenvironmental factors, resulting in an organ microenvironment that is immune tolerant and permissive to tumor growth. However, the current literature on the adrenal gland immune microenvironment is limited, underscoring the need for better understanding of the immunobiology of this critical endocrine organ. Thus, the current scarcity of scientific studies on this topic is a novel opportunity to investigate and develop innovative treatment strategies for adrenal solid cancer metastases. In this literature review, we summarize the available data published on the immunobiology of the adrenal gland and the potential local immune mechanisms that may be contributing to the adrenal gland’s role in promoting resistance to otherwise breakthrough immunotherapy treatments.

## 1. Introduction

The adrenal glands play a crucial function in the host endocrine system. The adrenal cortex is the primary site of steroidogenesis, which produces mineralocorticoids, glucocorticoids, and androgens. Likewise, the adrenal medulla has the critical role of producing catecholamines (epinephrine and norepinephrine). These hormones are key in multiple systemic processes, including the body’s stress response, immune homeostasis, metabolism, and tissue repair [1]. However, less is known about how these hormones regulate the biology of the adrenal gland itself, which is critical for local metabolic and immune homeostasis. In addition, the adrenal glands are an important site of solid tumor metastasis. Metastatic spread to the adrenal gland is predominantly observed from lung, kidney, melanoma, and colorectal cancers [2]. Notably, adrenal metastases are found in up to 27% of cancer patients at autopsy [3,4,5]. Although some studies support the resection of solitary adrenal metastasis with favorable oncologic outcomes [6,7,8], adrenal metastases are more commonly observed in the setting of disseminated cancer, making surgical resection contraindicated. In the setting of widely metastatic disease, the overall prognosis for adrenal metastases remains poor [9,10]. Importantly, metastatic lesions to the adrenal gland have also been shown to have poor response to immunotherapy [6,11,12,13]. For example, melanoma patients with adrenal metastases (a cancer type for which more robust data are available) have been shown to have poorer response rates to immune checkpoint inhibitors (ICIs) (16%) compared to patients without adrenal metastases (55%) or patients with non-adrenal metastatic sites (22%) [13]. In fact, this disparity in the efficacy of ICI treatment for adrenal metastases has led to speculation that the adrenal may potentially act as an immunosuppressive “sanctuary site” that is permissive to tumor growth and resistant to immunotherapy treatments, specifically ICIs. Therefore, the unique and under-researched immunobiology of the adrenal gland is an opportunity to investigate potentially novel mechanisms of adrenal immune tolerance, leading to the future development of innovative treatment strategies. However, the current literature on the adrenal microenvironment and its potential role in the growth of metastatic lesions is sparse. In this review, we will summarize the available published data on this important topic and discuss areas for future investigation into the mechanisms that may be contributing to adrenal gland potentially serving as a sanctuary site for tumor progression

## 2. Metastasis to the Adrenal Glands

Metastatic lesions are the most common malignant tumor found in adrenal glands. Common sources of metastases to the adrenal glands include the lungs, kidneys, gastrointestinal tract, and skin [14]. Historically, these lesions were discovered following symptomatic presentation in advanced cancer patients. More recently, adrenal metastases are more commonly identified incidentally on advanced cross-sectional imaging studies like CT, MRI, or PET/CT [15,16]. Metastatic lesions are now typically asymptomatic due to earlier detection when they are smaller, but in infrequent cases, bilateral lesions can cause adrenal insufficiency [17].

Overall, isolated metastases to the adrenal gland are rare and reported frequences depend on the primary tumor type. A single institution series of 94 adrenalectomies performed for isolated adrenal metastases reported that 70% of lesions were metachronous metastases occurring greater than 6 months after resection of the primary tumor [18]. The most common primary cancer diagnosis was non-small-cell lung cancer (NSCLC), followed by renal cell and colon cancers, consistent with common primaries for adrenal metastases. In NSCLC, the frequency of isolated adrenal metastasis varies across studies, with some having reported the rate of isolated adrenal metastases to be between 1.6 and 3.5% [19], while other reviews have cited a higher rate of 1–10% [20,21,22]. However, these figures are not readily generalizable across other malignancies. Overall, descriptions of isolated adrenal metastases are frequently limited to case reports including patients with hepatocellular carcinoma [23,24,25], prostate adenocarcinoma [26], and ovarian adenocarcinoma [27]. In settings of isolated adrenal metastases, aggressive local therapy with resection or ablative therapies are well tolerated and associated with favorable long-term outcomes. For example, Chen et al. analyzed 12 colorectal cancer patients with unilateral adrenal metastasis and found a median survival of 38.2 months following surgical resection of the affected adrenal gland [9]. Given the rarity of isolated adrenal metastases and the fact that diffusely metastatic disease involving the adrenal gland is generally managed according to primary tumor, the true incidence of isolated adrenal metastases across all primary tumors is unknown, and centralized reporting in registries has historically been lacking.

While the primary pathway for metastasis to the adrenal gland is hematogenous, it can also occur lymphatically or via local invasion. Studies on metastatic renal cell carcinoma (RCC) spread have found rates of adrenal metastases to range from 2% to 9% [28,29]. Furthermore, an autopsy study with 1828 RCC patients observed ipsilateral adrenal metastases in 2.5% of patients and contralateral adrenal metastases in 0.7% of patients [30]. Bilateral adrenal metastasis was exceptionally rare [31]. Importantly, a postmortem analysis of 216 patients with advanced-stage melanoma found adrenal metastases in 47% of cases, suggesting that occult adrenal metastases are significantly more common than clinically detectable metastases [32]. Breast cancer also can metastasize to the adrenal glands [33]. In a 1993 study evaluating 359 patients with invasive lobular carcinoma and 2246 patients with invasive ductal carcinoma, adrenal metastases were identified in 0.6% and 0% of cases, respectively [34]. This study demonstrated the rarity of these metastases and suggests that invasive lobular carcinoma may be more likely to metastasize to the adrenal glands than invasive ductal breast carcinoma. Other uncommon sources of adrenal metastasis include the esophagus, liver, bile ducts, and thyroid [35,36,37]. Ultimately, adrenal metastasis can occur from a wide array of primary cancer types, and, overall, it is the 4th most common site of metastasis [38].

## 3. Surgery, Chemotherapy, and Radiotherapy for Adrenal Metastases

Treating the underlying primary cancer is considered the first step in treating metastasis to adrenal gland. However, studies have shown that surgical resection with adrenalectomy can be used to address isolated adrenal metastasis with substantial benefits for disease-free interval [39]. Reports regarding adrenalectomy have shown 5-year survival rates of above 25% [6,18,40]. However, the benefit of adrenalectomy for metastases appears to critically depend on features of the primary tumor such as a favorable disease-free interval (DFI) and the presence of a solitary or oligometastatic lesion [41]. In contrast, historical series suggest that isolated adrenal metastatic lesions that undergo chemotherapy have poorer survival on the order of 6 to 9 months [42,43]. Modern stereotactic body radiation therapy (SBRT) for adrenal metastases has also emerged as a noninvasive ablative method in oligometastatic patients [44], but a study by Lutscher et al. showed that patients treated with adrenalectomy showed a higher 2-year survival rate compared to SBRT (83.3% vs. 40.2%) [45]. These results reinforce the idea that surgery is the preferred local treatment for patients with isolated adrenal metastasis. Despite effective local treatment for solitary adrenal metastases, the more common presentation for adrenal metastasis is that of disseminated disease. Outcomes from previous studies with adrenal metastases are summarized in Table 1. Cancer treatment for patients with diffuse metastasis remains a pivotal challenge in the oncology field; however, the innovation of ICI has drastically changed the treatment landscape.

## 4. Immunotherapy for Adrenal Metastases

ICIs have revolutionized cancer treatment options for patients with metastatic disease. The response rates to ICIs range from 40–70% in cancers like melanoma and Hodgkin’s disease [50,51] and 20–25% in non-small-cell lung cancer and renal cell carcinoma. Similarly, ICI treatment has resulted in a 24–40% decrease in mortality risk relative to standard chemotherapy in patients with lung cancer [52]. Despite the otherwise encouraging response rates to ICIs overall, metastatic lesions in the adrenal gland tend to have a poor response to PD-1 blockade and other ICIs. Studies suggest that the adrenal gland’s endogenous production of corticosteroids is linked to an immunosuppressive local microenvironment, allowing metastatic cells to escape immunosurveillance [13]. Currently, adrenal metastasis remains a consistently unfavorable site in the ICI era. For example, Billon et al. performed a phase II GETUG-AFU-26 NIVOREN study in metastatic renal cell carcinoma patients receiving nivolumab who previously underwent unsuccessful therapy with a tyrosine kinase inhibitor (TKI), which showed that adrenal metastasis was associated with lower objective response rates (12.5% vs. 23.2%, *p* = 0.005), shorter 6-month progression-free survival (PFS) (27.2% vs. 36.6%, *p* = 0.008), and inferior 12-month survival (64% vs. 71.1%, *p* = 0.0006) compared to metastases in other locations [47]. Similar patterns of site-specific resistance have been described in melanoma, where adrenal lesions often progress despite regression or stability at non-adrenal sites following anti–PD-1 or anti–cytotoxic T-lymphocyte associated protein 4 (CTLA-4) treatment. Borgers et al. retrospectively analyzed 68 melanoma patients with adrenal metastases, observing response rates for the adrenal metastasis group with the lowest response rates to ICIs (16%) compared to patients without adrenal metastases (55%) or patients with non-adrenal metastatic sites (22%) [13]. Cohen et al. also reported a case series of patients with metastatic colorectal cancer who were treated with ICIs and exhibited disease progression limited to the adrenal glands [48]. Another case series of melanoma and uterine carcinosarcoma patients treated with pembrolizumab further reinforces this pattern, demonstrating adrenal lesions that either persist or newly arise as the sole site of progression in the setting of otherwise durable systemic responses, providing further evidence of putative adrenal-gland-specific ICI resistance mechanisms [11]. Collectively, these studies reinforce the impression that the adrenal glands are a site of relative resistance to ICIs across tumor types and should be regarded both as an adverse prognostic feature and as a candidate for early, aggressive local management within multimodal immunotherapy strategies.

## 5. Mechanisms of Immunotherapy Resistance

The response rates to ICIs vary significantly by primary tumor type [53]. For example, across solid tumors, anti–PD-1/PD-L1 monotherapy yields objective responses rates in roughly 20% of unselected patients, with higher response rates in hypermutated or PD-L1–high cancers [54,55]. Notably, responses are also higher in particularly immunogenic malignancies, reaching 45–60% in melanoma and microsatellite instability (MSI)-high tumors [56]. Anti-PD-1 therapy also shows responses exceeding 80% in chemotherapy-refractory Hodgkin lymphoma treated with anti–PD-1 therapy [57]. Yet, even high-tumor-mutational-burden tumors across 27 tumor types show substantial primary and acquired resistance, suggesting additional tissue-specific factors [55].

Most patients who do not benefit from immune checkpoint inhibitors fail because of primary or innate resistance, but secondary or acquired resistance remains an important mechanism as well. Immunotherapy resistance can be broadly divided into tumor-intrinsic and tumor-extrinsic processes and may occur at the initial, adaptive, or acquired stages of response [58]. Tumor-intrinsic mechanisms include loss of antigen expression, defects in antigen presentation, oncogenic programs that exclude or hamper T cells, and impaired sensitivity to effector cytokines. Good examples of intrinsic resistance include mixed loss-of-function mutations in the interferon-receptor-associated kinases Janus kinase 1 (JAK1) or Janus kinase 2 (JAK2), together with truncating mutations in the antigen-presentation component β2-microglobulin (B2M), which have been linked to acquired resistance to PD-1 blockade in melanoma [59]. Similarly, in human metastatic melanoma, activation of the WNT/β-catenin pathway correlates with absence of a T cell gene signature and resistance to anti-PD-L1/anti-CTLA-4 therapies [60].

Although predictive markers of response to ICIs remain an unmet need in the field, there are several established biomarkers of ICI response. For example, one major predictor for ICI therapy is PD-L1 expression in the tumor microenvironment [61]. Patients can be clinically assessed for potential anti-PD-1/anti-PD-L1 therapies based on the tumor proportion score (TPS) of tumor cells with membranous PD-L1 expression [62]. Transcriptomic analysis has also shown that the expression of PD-1 correlates with prolonged survival in patients who received ICIs [63]. Notably, PD-L1 positivity ranges from 24 to 60% in NSCLC [64] and is approximately 28% in metastatic papillary renal cell carcinoma [65]. Another popular predictor of ICI response is mismatch repair-deficient (dMMR) tumors with high microsatellite instability (MSI), which is clinically validated as a biomarker for ICI treatment in solid tumors [66]. Tumor mutation burden (TMB) refers to the total number of mutations in a tumor’s DNA, and it is also a validated predictive biomarker for ICI response, with high-TMB tumors reproducibly demonstrating better outcomes [67]. Since ICIs make use of pre-existing host immune responses, CD8^+^ T cell replete tumor immune microenvironments have also been shown to be a predictor for improved response and survival [68,69]. However, among these identified biomarkers of ICI response, there have been very few studies that pertain to the adrenal gland tumor microenvironment. One study by Pollack et al. evaluated PD-L1 staining in 17 formalin-fixed paraffin embedded adrenal samples and found that 15 samples had no PD-L1 staining and 2 samples only had <1% focal cytoplasmic staining [70]. Another retrospective study of PD-L1-positive (PD-L1 > 1%) patients with adrenal metastases treated with immunotherapy observed that PD-L1 had a paradoxical favorable predictive effect and an unfavorable prognostic effect [71]. Nevertheless, the potential role of ICI predictive biomarkers remains unclear in the context of the adrenal gland and this subject requires more formal investigation.

Another important concept in ICI resistance is acquired (secondary) resistance [72]. Overall, the mechanisms of acquired ICI resistance are not well understood. However, one hypothesis is that tumors can be affected by ICIs at the genomic level. For example, Anagnostou et al. showed that the mutational landscape of NSCLC patients evolved pre- and post-ICI treatment [73]. The dysregulated expression of various biomarkers in response to ICIs has also been linked to mechanisms of acquired resistance. For example, the loss of phosphatase and tensin homolog (PTEN) expression has been shown to result in diminished T cell infiltration and increased ICI resistance [74] and this mutation has been demonstrated in a non-responding lesion in a patient with metastatic uterine leiomyosarcoma treated with ICIs [75]. Potential mechanisms of acquired ICI resistance in the adrenal gland are currently unknown, as even innate ICI resistance in the adrenal gland is a topic that still requires future study.

Broadly speaking, responses to ICIs often appear dualistic: most non-responders progress at a pace like the natural history of their disease, whereas a subset achieves profound and sometimes prolonged control. However, extended follow-up of clinical trial cases has shown late relapse in initially responding patients, and the increasing frequency of this observation underscores the importance of acquired resistance even in tumors that are initially ICI-sensitive [72]. While classic innate and adaptive mechanisms of ICI resistance are important to consider, tissue-specific factors may play a more important role than previously thought in tumor-extrinsic immunotherapy resistance. One example of a tissue-specific resistance factor that has been investigated is in the liver, which has been demonstrated to restrain ICI efficacy via macrophage-mediated T cell elimination [76]. Another study showed that liver metastases displayed a genomic landscape with lower immune activation and infiltration levels, reinforcing the concept of organ-specific ICI resistance [77]. Yet, overall, tissue-specific factors (such as the factors in the adrenal gland microenvironment) are a comparably notable knowledge gap in the cancer immunology field and require further scientific study.

## 6. The Effect of Adrenal Endogenous Hormone Production on the Immune Microenvironment

**Figure 1:**
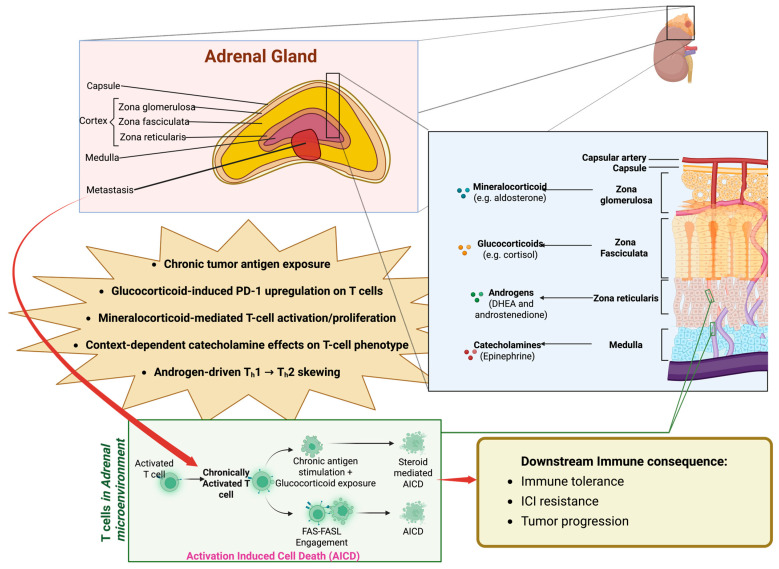
The adrenal microenvironment, with potential mechanisms of immunosuppression for further investigation, including metastatic tumor deposits providing a source of persistent antigen stimulation, which could then promote T cell activation-induced cell death in the steroid hormone-rich adrenal domain (Created in BioRender. Karimzadeh, M. (2026) https://BioRender.com/0qyvakp, accessed on 22 January 2026).

The adrenal-specific pattern of metastatic resistance to ICIs suggests that features of the adrenal immune microenvironment such as chronic glucocorticoid exposure, local stromal and vascular niches, or distinct myeloid and lymphoid composition may attenuate anti-tumor effects following ICIs [78,79,80,81]. However, pre-clinical and translational studies have not systematically profiled the immune microenvironment of the adrenal glands nor adrenal metastases in ICI-treated patients; so, mechanistic explanations for adrenal ICI resistance remain largely inferential rather than experimentally demonstrated. This gap underscores the need for dedicated translational studies of the adrenal gland and adrenal metastases to determine whether their relative resistance to ICIs is driven by local microenvironmental factors. Nevertheless, the most unique adrenal-specific factor that may be influencing ICI responses is the adrenal glands’ endogenous production of different steroid hormones (glucocorticoids, mineralocorticoids, and androgens) and catecholamines (epinephrine and norepinephrine) (Figure 1).

Glucocorticoids (most notably cortisol in humans) are produced by the cells in the zona fasciculata of the adrenal cortex and have a critical role in glucose metabolism, systemic immunity, and stress responses. The local hormone concentrations of glucocorticoids are of particular interest in the adrenal microenvironment because glucocorticoids have been demonstrated to have significant effects on various immune cell types. For example, glucocorticoids are known to inhibit T cell activation, proliferation and cytokine production [82,83]. Likewise, studies have shown that glucocorticoids can reprogram cytotoxic CD8^+^ T cells and natural killer (NK) cells by upregulating inhibitory receptors such as PD-1 and CTLA-4 while simultaneously reducing expression of effector molecules like perforin, interferon-γ (IFN-γ), and granzymes [84], which collectively results in weakened killing of tumor cells. In contrast, T helper 2 (Th2) cells (characterized as CD4^+^ T cells that classically express interleukin 4 (IL-4), interleukin 5 (IL-5), and interleukin 13 (IL-13) to promote antibody production and response to parasites and antigens) [85] have been shown to express cytochrome P450 side family 11 subfamily A member 1 (CYP11A1), enabling de novo steroidogenesis from cholesterol, which inhibits further T-helper proliferation and influences B cell class switching, contributing to broader immune dampening [86,87].

Glucocorticoids also have various effects on innate immunity. Endogenous glucocorticoids are known to promote mobilization of neutrophils from bone marrow into circulation [88]. However, glucocorticoids also exert control on the physical distribution of neutrophils in the body. They downregulate expression of L-selectin (CD62L), an adhesion moecule that allows for neutrophil rolling along vessel endothelium prior to penetrating into tissue. Another contributing factor to L-selectin downregulation is Annexin-1, which is induced by glucocorticoid signaling [89]. L-selectin downregulation leads to functional suppression of neutrophils by impairing their migration into peripheral tissues [90]. Ultimately, this has an anti-inflammatory effect. Moreover, the effects of glucocorticoids on macrophages are more related to effector function rather than navigation through tissue. Glucocorticoids appear to downregulate the pro-inflammatory M1 phenotype of macrophages, instead favoring macrophage differentiation toward the anti-inflammatory M2 phenotype, demonstrating another downregulation of inflammatory activity [91]. The anti-inflammatory effect of glucocorticoids on immune cells such as neutrophils and macrophages is of particular importance in the adrenal glands, where endogenous glucocorticoids are produced. The presence of glucocorticoids in the adrenal cortex could have a role in downregulating immune responses against local tumors, providing a permissive environment for tumor growth.

While local glucocorticoid concentrations may significantly affect the adaptive and innate immunity of the adrenal gland, systemically administered glucocorticoids also have crucial effects on tumor-immune interactions. Furthermore, the administration of systemic glucocorticoids has been linked with poorer outcomes in various cancer types treated with ICIs. A meta-analysis of 4045 ICI-treated patients across 16 studies found that systemic steroid use was associated with increased risks of death and disease progression, reinforcing the potential negative prognostic implications of exogenous glucocorticoids in this setting [92]. For example, in advanced NSCLC, Arbour et al. showed that patients receiving ≥10 mg prednisone-equivalent at the start of PD-1/PD-L1 therapy had significantly lower response rates and markedly shorter PFS and overall survival (OS) than steroid-free patients [93]. Given that this was a retrospective analysis, it remains possible that the association of glucocorticoid use with worse outcomes following ICIs was confounded by the underlying indications which prompted steroid use, thereby biasing the data. In fact, the effect of systemic glucocorticoids on response rates and oncologic outcomes with ICI treatment remains controversial, and other studies, such as one by Horvat et al., showed that corticosteroid usage did not affect the overall survival and time to treatment failure for melanoma patients treated with ipilimumab [94]. Moreover, steroids have been used to manage immune-related adverse events (irAEs) from ICIs, and studies have shown that their usage in treating irAEs does not seem to directly impair clinical benefits [56,95,96,97,98,99].

Mineralocorticoids (aldosterone) are produced in the zona glomerulosa of the adrenal cortex and regulate fluid and electrolyte balances in the body by promoting the retention of sodium and the excretion of potassium [100]. The mineralocorticoid receptor (MR) is expressed in various tissue types, including the kidney, blood vessels, heart, and central nervous system [101]. Additionally, recent evidence has further linked MR more broadly in immune biology with studies identifying MR expression on monocytes, macrophages, dendritic cells, and T cells [102]. However, unlike glucocorticoids, which commonly demonstrate immunosuppressive effects, aldosterone signaling through MR has been shown to be pro-inflammatory. For example, aldosterone has been shown to promote autoimmune-related damage through dendritic-cell-mediated activation of CD8^+^ T cells [103]. Additionally, aldosterone has been associated with decreased activation of suppressive regulatory T cells (Treg), resulting in greater inflammatory effects [104]. Furthermore, MR knockout in cultured CD8^+^ T cells was shown to suppress IFN-ɣ expression, showing the link between aldosterone signaling and immunostimulatory cytokine secretion [105].

Precursor androgens (dehydroepiandrosterone (DHEA), androstenedione) are produced in the zona reticularis of the adrenal cortex and are subsequently converted to testosterone in the testicles and estrogen in the ovaries [106]. Androgens regulate secondary sex characteristics and play an important role in development [107]. Studies have also shown that androgens shape immunity at multiple levels. Androgen receptors (ARs) are found on a myriad of immune cells, including neutrophils, monocytes, and B and T lymphocytes [108]. Overall, evidence points toward an immunosuppressive role of androgens in several different immune cell types [109]. For example, in stimulated macrophages in vitro, testosterone has been shown to suppress tumor necrosis factor alpha (TNF-α) and interleukin 1B (IL-1B), supporting a potential anti-inflammatory role. Regarding T cells, androgen administration was shown to have a negative impact on thymic growth in mice, and vice versa with androgen deficiency [110]. This suggests a possible effect on subsequent T cell production and differentiation. Androgen signaling in both tumor and myeloid cells has been linked to increased tumor burden in preclinical studies [111]. For CD4^+^ T cells, androgens have been shown to reduce T helper 1 (Th1) differentiation and IFN-γ production while promoting Treg expansion and forkhead box P3 (FOXP3) and interleukin 10 (IL-10) expression, shifting the balance toward an anti-inflammatory phenotype [111,112]. In contrast, genetic or pharmacologic inhibition of AR in CD8^+^ T cells appears to restore stem-like and effector populations and enhances checkpoint inhibitor efficacy in murine models [113,114,115]. While androgens have demonstrated various mechanistic effects on immune cells, some studies have also shown that there may be a favorable effect of androgen signaling on ICI responses in obese males compared to obese females [116,117]. In contrast, a recent study by Guan et al. showed that AR activity in T cells limits the efficacy of ICIs [113]. Nevertheless, the mechanisms underlying potential sex-specific differences in ICI responses remain unclear, but likely are linked to the various effects that estrogen and testosterone can have on immune cell function [118]. Another potential mechanism is sex-based dimorphism in the tumor microenvironment, as seen in an NSCLC study that showed a higher immune infiltration in tumors among females compared to males [119].

Lastly, catecholamines are produced in the adrenal medulla, which is composed of neuroendocrine cells called chromaffin cells that produce epinephrine (adrenaline), norepinephrine (noradrenaline), and dopamine [120]. These catecholamines are associated with the sympathetic nervous system and result in regulation of the body’s flight-or-fight response, basal metabolic rate, heart rate, blood pressure, and smooth muscle dilation/constriction. Catecholamines have also been shown to modulate immune function. Epinephrine and norepinephrine signal through binding to the α-adrenergic and β-adrenergic family of receptors. Notably, both innate and adaptive immune cells have been shown to also express adrenergic receptors, which allows for direct interaction with catecholamines [121]. Generally, catecholamines have demonstrated an immunosuppressive effect. For example, the β2-adrenergic receptor has been shown to suppress the secretion of inflammatory cytokines in macrophages and dendritic cells [122]. In addition, norepinephrine binding to the β2-adrenergic receptor can induce the rapid secretion of IL-10, which is a cytokine that has anti-inflammatory effects [123]. Catecholamines have also displayed the ability to modulate antigen presentation. For example, adrenergic signaling has been shown to decrease cross-presentation in dendritic cells, which results in suppression of CD4^+^ T cell activation [124].

## 7. The Immune Milieu of the Adrenal Gland Microenvironment

Classically, studies of the immunology of the adrenal glands have focused on the effects of adrenal gland hormones on systemic immunity and immune responses. In contrast, there have been very few publications regarding the immune milieu within the adrenal gland itself. However, the limited studies that exist on this topic have shown that the adrenal gland microenvironment may harbor more immune cells than expected and some single-cell and histological analyses indicate that the normal adrenal microenvironment is macrophage-dominated, with abundant tissue-resident and infiltrating macrophages and substantial T cell populations, including lymphocytes localized to the zona glomerulosa [125,126,127]. Additionally, recent studies have demonstrated that adrenal gland macrophages may restrict steroidogenesis through transforming growth factor beta (TGF-β) and triggering receptor expressed on myeloid cells 2 (Trem2) [128]. Overall, the limited number of studies regarding immune cells in the adrenal gland shows that this subject requires further scientific investigation, especially in the context of cancer immunobiology. Another aspect that may influence the adrenal immune milieu and warrants further research is the role of metabolic reprogramming. Research has shown that proliferating and metastasizing tumors may upregulate multiple pathways in lipid, amino acid, and glucose metabolism, such as the tricarboxylic acid cycle [129]. This increased metabolism can result in a more hypoxic and acidic environment with decreased nutrients and increased metabolic byproducts, creating an overall immunosuppressive setting that can inhibit anti-tumor immune responses [130]. While metabolic reprogramming has been more studied in primary adrenocortical carcinoma, with these tumors showing increased biomarkers of anaerobic pathways compared to adrenal adenomas [131], the subject of metabolic reprogramming is still under-researched with regard to the growth of metastatic lesions in the adrenal gland. Therefore, further investigation is needed to see how the combination of endogenous hormone production in the adrenal gland may be affecting metabolic reprogramming and potentially contributing to an immunosuppressed microenvironment.

Additionally, another important area of investigation is the effect of stroma-immune crosstalk in the adrenal gland microenvironment. Stroma cells such as fibroblasts, epithelial cells, and endothelial cells play a role in the immune responses of various organs. For example, epithelial cells and fibroblasts in the lungs secrete various cytokines that result in the increased recruitment and activation of immune cells after pulmonary infection [132]. While little is known regarding stromal effects on the adrenal gland itself, research has shown the importance of stroma-immune interactions in various cancers that commonly metastasize to the adrenal gland (such as NSCLC and RCC), which suggests its importance in metastatic progression within the adrenal domain. For example, there are immune-poor niches within the NSCLC tumor microenvironment that are reinforced by a high level of stromal complexity, which keeps lymphocytes away from tumor sites, allowing tumors to evade immune detection [133]. Likewise, RCC tumor cells secrete cytokines that activate carcinoma-associated fibroblasts, which, in turn, help to recruit immunosuppressive cells such as Tregs [134] and actively inhibit immune cell function through secretion of TGF-β and arginase-2 (ARG2), facilitating tumor progression [135]. However, stroma-immune crosstalk in the adrenal gland itself is still unclear and is an important topic for future study. Another crucial factor affecting the adrenal gland immune milieu is the adrenal vasculature itself. The adrenal gland is a highly vascularized organ with many fenestrated sinusoids that facilitate secretion of steroid hormones [136]. Additionally, adrenocorticotropic hormone (ACTH) increases production of vascular endothelial growth factor (VEGF) in the adrenal gland [137]. While the adrenal vasculature plays a fundamental role in adrenal gland function and hormone secretion, it likely also plays a role in the recruitment and function of immune cells. Overall, the immune milieu of the adrenal gland remains an understudied topic and requires further investigation.

## 8. Immune Privilege in the Adrenal Gland Microenvironment

The adrenal gland has been previously speculated to be an immune privileged organ [138,139], although detailed studies supporting this hypothesis are lacking. Immune privilege is the phenomenon where certain organs tolerate exposure to new antigens without activating a typical inflammatory response. This concept originated from the observation that tissue grafts are more easily incorporated in certain sites in the body, such as the eyes, compared to other sites which exhibit acute and chronic rejection reactions [140]. Immune privilege is hypothesized to serve an evolutionary purpose for protecting vital organs from the damaging effects of inflammation and the development of autoimmunity [141,142]. While immune privilege can be a protective mechanism against the undesirable effects of inflammation, the unintended consequence is that these tissue sites may also be more susceptible to viral infection [143] or tumor growth [144].

Examples of immune privilege include the eyes, the testicles, the pregnant uterus, and the central nervous system [145,146]. Previously, immune privilege was thought to be due to a scarcity of immune cells, which may be restricted from tissue entry due to physical blood–tissue barriers. However, the more contemporary understanding of immune privilege is that it is the result of organ-specific mechanisms that modulate and suppress immune cell activity [146,147]. It has become more apparent that T cells are the key to these unique mechanisms of immune privilege. For example, studies have shown that immune privilege in the eye is influenced by the functional expression of FasL, leading to apoptosis of Fas+ T cells [148]. In contrast, immune privilege in the pregnant uterus is affected by the lack of human leukocyte antigen A (HLA-A) and human leukocyte antigen B (HLA-B) expression on placental villi and the extra villous trophoblast, resulting in these cells being hidden from detection by maternal T cells and reducing the risk of fetal loss [149]. Testicular immune privilege may also be influenced by the balance of T cells subsets, as studies have shown that mouse testicles with implanted allografts have altered T cell subpopulations, with increased destruction of memory T cells and increased recruitment of Tregs [150,151].

Due to the scarcity of research on this subject, the question of adrenal immune privilege remains highly speculative with limited evidence. However, this topic provides a promising avenue for future scientific investigation. Possible mechanisms of immune privilege that could be investigated include apoptosis or activation-induced cell death of immune cells, immune suppression from endogenous steroid hormone secretion, and the interaction of anti-inflammatory cytokines such as TGF-β and IL-10 or cells like Tregs and myeloid derived suppressor cells (MDSCs). Nevertheless, the clinical observation that adrenal metastases show resistance to ICIs still supports that the adrenal glands are indeed a sanctuary site for tumor growth [152], and the lack of studies on this topic represents a prime opportunity for further scientific investigation into potential adrenal immune privilege.

## 9. Conclusions

In conclusion, the adrenal gland is an important site for metastasis. While surgery has been shown to be effective in solitary metastatic disease, adrenal metastases of multiple primary tumor types have demonstrated poor response to ICIs. There is currently no established explanation behind this tendency, although the steroid-producing adrenal gland microenvironment is heavily suspected to play a role in the mechanisms of adrenal gland resistance to ICIs (Figure 1). Thus, there is still a significant gap in basic research regarding the mechanisms of immune suppression in the adrenal gland, and the poor efficacy of immunotherapy in the treatment of adrenal metastasis creates an innovative opportunity to investigate the unique mechanisms of adrenal immune tolerance, which could lead to the future development of novel treatment strategies.

## Figures and Tables

**Table 1:** Outcomes summary for previous studies on adrenal metastases.

Study	Primary Tumor	Patients [*n* = ]	Treatment	DFI (mo)	OS	OS	OS	PFS	RR %
					Median	1 yr %	2 yr %	Median	
Chen et al. 2024 [9]	Colorectal	13	Surgery	3.4–69.3 mo	38.2 mo	NR	NR	NR	NR
Plichta et al. 2017 [44]	Various	10	SBRT	NR	9.9 mo	NR	NR	3.4 mo	NR
Lütscher et al. 2024 [45]	Various	41 (48 adrenal metastasis)	27/41 SBRT (65.9%) vs. 14/41 Surgery (34.1%)	NR	97 mo (surgery) vs. 15 mo (SBRT) (*p* = 0.031)	83.9% (surgery) vs. 67.0% (SBRT)	83.9% (surgery) vs. 40.2 (SBRT)	8 mo (surgery) vs. 4 mo (SBRT) (*p* = 0.223)	NR
Chen et al. 2020 [46]	Various	1006	SBRT + concurrent immunotherapy and chemotherapy	NR	NR	66%	42%	NR	54.60%
Billon et al. 2024 [47]	Kidney	151	Immunotherapy (after initial TKI)	NR	NR	64% (adrenal metastases) vs. 71.1% (other metastases)	NR	NR	12.5% (adrenal metastases) vs. 23.2% (other metastases)
Borgers at al 2021 [13]	Melanoma	68	Immunotherapy	NR	3.1 yrs (adrenal metastases)	NR	NR	NR	16% (adrenal metastases) vs. 55% (no adrenal metastases) vs. 22% (other metastases)
Hatano et al. 2020 [39]	Various	25	Surgery	NR	63 mo	NR	NR	14 mo	NR
Cohen et al. 2021 [48]	Colorectal	5	Immunotherapy	NR	NR	NR	NR	NR	80%
Jajodia et al. 2022 [49]	Kidney	21	Immunotherapy	NR	NR	NR	NR	NR	35% (RECIST 1.1) and 50% (iRECIST).

mo = months; yrs = years; PFS = progression-free survival; RR = response rate; SBRT = stereotactic body radiation therapy; DFI = Disease free interval; NR = not reported; TKI = tyrosine kinase inhibitor; RECIST = Response Evaluation Criteria in Solid Tumors; iRECIST = Immune-related Response Evaluation Criteria in Solid Tumors.

## Data Availability

No new data were created or analyzed in this study. Data sharing is not applicable to this article.

## Abbreviations

The following abbreviations are used in this manuscript:

ICI(s)	Immune checkpoint inhibitor(s)
PD-1	Programmed cell death protein 1
PD-L1	Programmed death-ligand 1
CT	Computed tomography
MRI	Magnetic resonance imaging
PET	Positron emission tomography
RCC	Renal cell carcinoma
SBRT	Stereotactic body radiation therapy
DFI	Disease free interval
PFS	Progression free survival
CTLA-4	Cytotoxic T-lymphocyte associated protein 4
MSI	Microsatellite instability
NSCLC	Non-small-cell lung cancer
TMB	Tumor mutation burden
TPS	Tumor proportion score
NK	Natural killer
IFN-γ	Interferon gamma
IL	Interleukin
irAEs	Immune-related adverse events
MR	Mineralocorticoid receptor
AR	Androgen receptor
TGF-β	Transforming growth factor beta
Trem2	Triggering receptor expressed on myeloid cells 2
HLA	Human leukocyte antigen
Treg	Regulatory T cell
MDSC	Myeloid derived suppressor cell
